# Text Reminders in Colorectal Cancer Screening (TRICCS): Protocol for a randomised controlled trial

**DOI:** 10.1186/s12889-016-2733-6

**Published:** 2016-01-25

**Authors:** Yasemin Hirst, Robert Kerrison, Lindsay C. Kobayashi, Nicholas Counsell, Natasha Djedovic, Josephine Ruwende, Mark Stewart, Christian von Wagner

**Affiliations:** 1 Health Behaviour Research Centre, Department of Epidemiology and Public Health, University College London, 1-19 Torrington Place, London, WC1E 7HB UK; 2 Cancer Research UK & UCL Cancer Trials Centre, Cancer Institute, University College London, 90 Tottenham Court Road, London, W1T 4TJ UK; 3 Bowel Cancer Screening Hub - London, Northwick Park & St. Mark`s Hospitals, Watford Road, Harrow, Middlesex HA1 3UJ UK; 4 NHS England London Region, Southside, 105 Victoria Street, London, SW1E 6QT UK

**Keywords:** Colorectal cancer screening, Faecal occult blood test, Text-message, Mobile health, London

## Abstract

**Background:**

Screening with the guaiac faecal occult blood test (gFOBt) is associated with improved colorectal cancer (CRC) survival, and is offered biennially to men and women aged 60–74 years in England’s national Bowel Cancer Screening Programme (BCSP). Uptake of the gFOBt is low, with only 54 % of the eligible population completing the test. Text-message reminders could improve uptake of gFOBt.

**Methods/design:**

This paper describes the protocol for a randomised controlled trial, which will examine the effectiveness of a text-message reminder to promote uptake of gFOBt screening in the BCSP. Individual mobile telephone data from 180 general practices in London with existing mobile-health services will be linked to the national BCSP information system via a secure on-line network. All screening-eligible adults registered with a participating practice will be randomised, to receive either usual care (*N* = 1600) or usual care plus a text-message reminder to self-complete and return their kit eight weeks after their initial invitation (*N* = 1600). The primary outcome will be the proportion of individuals who return an adequately completed gFOBt kit within 18 weeks of the initial invitation. Differences in uptake between groups will be evaluated using a logistic regression analysis, adjusting for individual-level and area-level socio-demographic variables.

**Discussion:**

This will be the first large-scale randomised trial of a text-message reminder in a national screening programme for CRC. If effective, this study provides a cost-effective means to promote uptake of CRC screening in an organised programme.

**Trial registration:**

Current Controlled Trials ISRCTN70904476 (18/09/2015).

## Background

Colorectal cancer (CRC) is the fourth most common cancer in the United Kingdom [1] and the second leading cause of cancer mortality [2]. Screening is widely recommended for the early detection of CRC and is associated with improved survival outcomes [3–6]. In England, the National Health Service (NHS) runs an organised population-based screening programme for CRC (the ‘NHS Bowel Cancer Screening Programme’; BCSP) which offers biennial guaiac faecal occult blood testing (gFOBt) to men and women aged 60–74 years. However, uptake of the BCSP is the lowest of the three organised cancer screening programmes in England, with only 54 % of the age-eligible population taking up the screening test offer for CRC, compared with around 75 % in the breast and cervical cancer screening programmes [7–9].

Multi-component reminders, such as follow-up postal-reminders with scheduling assistance and successive rounds of telephone reminders are among the most effective methods to improve uptake of cancer screening services, yielding results over and above those of one-off postal reminders alone [10, 11]. In recent studies, researchers have also focused on the potential of mobile health (m-health) technologies to promote adherence to healthcare appointments [12, 13]. Several studies have highlighted that pre-appointment text-messages are not only an effective alternative for delivering reminders, but an acceptable and often preferred method of communication [14–16].

In the United States, a randomised trial examining the effectiveness of a multicomponent strategy to increase uptake of gFOBt-based CRC screening through community health centres found that text-message reminders, when used in conjunction with postal and automated telephone reminders, achieved uptake rates of 82 % (which compared favourably with the 37 % rates observed in the usual care group) [17]. In the context of organised screening in the UK, two recent studies found that text-message pre-appointment reminders were effective at increasing attendance at routine breast screening appointments [18, 19]; the effectiveness of text-message reminders to promote uptake of gFOBt screening for CRC in the NHS BCSP however, has not been examined yet [20]. Unlike pre-appointment reminders for the NHS Breast Screening programme [21] a reminder for CRC screening would act as an additional prompt to complete and return a gFOBt kit beyond the standard 4 week postal reminder.

This paper, therefore, describes the protocol for a randomised controlled trial (RCT) which will examine the effectiveness of adding a text-message reminder to the current NHS BCSP.

### Aims

The primary aim of this RCT will be to test the effectiveness (intention-to-treat analysis) of a text-message reminder to promote gFOBt uptake in the English BCSP (i.e. the total proportion of people adequately screened).

The secondary aim will be to examine the efficacy (per-protocol analysis) of the text-message reminder to promote gFOBt uptake among screening-eligible adults with a registered mobile number at their General Practice (GP).

## Methods/design

### Study design

This study will be a non-clinical RCT with two parallel arms (control & intervention). The control group will be invited to take part in the NHS BCSP as per the usual care pathway, while the intervention group will additionally receive a text-message reminder eight weeks into their episode. Text-message reminders will be sent via iPlato Ltd, Patient Care Messaging (PCM), a cloud-based communication platform specifically developed for health services [22], using the mobile telephone numbers stored on the clinical systems of participating GPs.

**Fig. 1:**
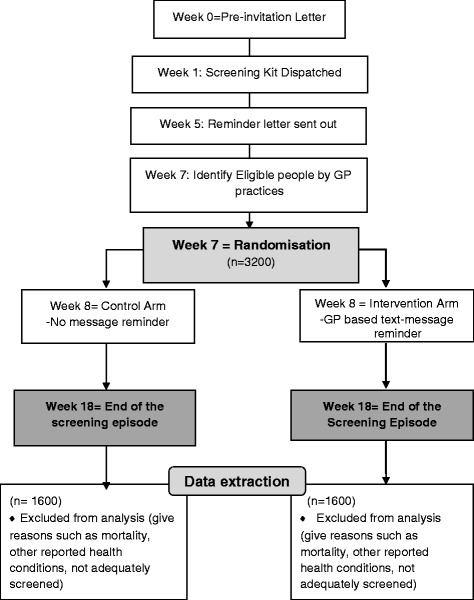
TRICCS Consort Flow diagram

The CONSORT diagram is shown in Fig. 1.

### Study setting

The RCT will be conducted in London, England, where uptake is below the national average, and will be conducted in collaboration with the London Bowel Cancer Screening Hub (hereafter referred to as ‘the Hub’) at St. Marks’ Hospital in Harrow, London. All GPs based within six pre-selected Clinical Commissioning Groups (CCGs: NHS organisations that manage patient care in GPs in defined geographical areas) will be invited to participate in the study. CCGs have been pre-selected on the basis that they are London-based and consistently achieve low CRC screening uptake rates (less than 50 %). Namely, we will invite GPs from the following CCGs: Greenwich, Croydon, Lewisham, West London, Hammersmith & Fulham, and Hounslow.

### Eligibility criteria

GPs will be eligible to take part in this study if: 1) they have been using text messaging services to communicate with their patients for a minimum of 12 months (this is to ensure that the individuals who will be included in this study have been given the opportunity to opt-out and/or consent to their GP contacting them via m-health services); and 2) they are not part of any other project or initiative to improve CRC screening uptake in London (to minimise confounding).

### Recruitment of general practices

All GPs located within the six aforementioned CCGs (*N* = 290) will be sent an invitation to participate in this study. The invitation will include: 1) a letter inviting the practice to participate in the study; 2) an information sheet outlining the rationale and design of the study and; 3) a research consent form for those practices which are interested in taking part in the study. The invitation letter will stipulate that a practice can only take part in the study if the practice meets the inclusion criteria and provides written consent. Similar studies examining the effectiveness of primary care-based materials to promote CRC screening have achieved response rates as high as 80 % when practices were offered an opt-out for their collaboration in research [23]. Due to the inclusion criteria and use of opt-in as the recruitment method, we expect a slightly lower response rate. We aim to recruit 180 practices (i.e. 62 % of all eligible sites).

### Invitation

#### The NHS Bowel Cancer Screening Programme Pathway (Usual Care Group)

At age 60, and then biennially up to and including the age of 74, adults registered with a GP in England (who have not explicitly opted out of the screening programme) are sent an invitation letter and information booklet by their local Hub notifying them that they will soon receive a test kit as part of a national screening programme for CRC. A gFOBt kit and instructions follow 8–10 days later; the individual is asked to collect samples from three consecutive bowel motions and to then return the completed kit to the Hub in a pre-paid envelope for processing. Repeat gFOBt kits are sent out where the returned kit has been completed improperly (a ‘spoilt kit’), where there is a technical failure in processing the kit or where there is an unclear result. A reminder letter is sent out after four weeks after the initial invitation. If thirteen more weeks pass with no response, the screening episode is closed (i.e. after 18 weeks) and the individuals practice receives a letter of notification regarding their non-participation in the programme. If the test results are abnormal, a referral is made to the local screening centre for further investigation.

#### Text-message Reminder (Intervention Group)

**Fig. 2:**
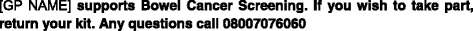
Text-message content in the intervention arm

Those who are randomised to the intervention group will be invited to participate in the NHS BCSP as per standard practice; and, additionally, receive a text-message reminder if they have not returned the test kit within eight weeks of receiving their initial invitation letter (i.e. three weeks after the non-response reminder letter). The text-message reminder will include the name of the person’s General Practice, the purpose of the text-message, and guidance on where to get more information (see Fig. 2). The content of the text-message is based on what was used as part of a previous General Practice endorsement study [23] and was further refined by a steering group involving patient representatives, GP Cancer Leads, Public Health England’s *Behavioural Insights Team* and the Hub.

#### Delivery of the text-message reminder

The text-message reminder will be delivered to individuals using the mobile numbers recorded on their GP’s Clinical Systems via iPlato Patient Care Messaging (PCM). iPlato is an Information Governance (IG) toolkit accredited m-health company [22], which provides text messaging services for healthcare providers. They will set up an encrypted cloud-based server, in which they will process patient identifiable information (NHS number, GP code, mobile number) for the secure delivery of the text-message to the recipient. iPlato will collate CRC screening related data (episode start date, kit returned date) from the HUB with patient mobile phone numbers from the GPs in the secure-encrypted cloud based server and will send text-message reminders to those randomised to the intervention and who have a ‘kit not received’ status on 8^th^ week of their on-going screening episode.

### Timeline

On the seventh week of each screening episode, all individuals invited to CRC screening from the participating GPs will be enrolled into the study and randomised to stay in the study for another eleven weeks until their screening episode is closed for gFOBt participation (excludes colonoscopy participation).

### Blinding & randomisation

Individuals registered at participating GP will not be informed that they are participants in a research study, and the study investigators will not know which individuals have been assigned to the intervention or control groups until the end of the trial when all data have been collected and anonymised. Randomisation of individuals to study groups will be handled by iPlato using simple pseudo-random allocation methods stratified by the preselected CCGs. Individuals will be randomised in a 1:1 ratio to either the intervention or control condition after 7 weeks of their screening episode irrespective of whether or not they have a mobile number registered on their GP’s Clinical System.

### Data processing and data collection

**Fig. 3:**
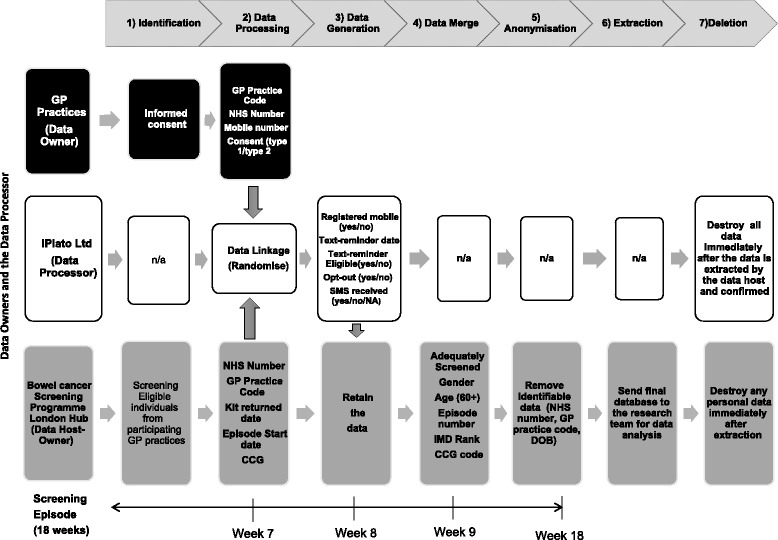
TRICCS Data Processing Diagram

An overview of the data processing and data collection processes is outlined in Fig. 3.

1. Identification
  On a weekly basis, the Hub will identify eligible patients who are seven weeks into a screening episode. Eligible individuals will be those who have not opted-out of screening and who are registered with participating GP practices.
2. Data Processing
  In the same week as the identification, iPlato will link data from the GP Clinical System with the Hub’s information system to randomise people into intervention and control groups. Data will be matched by via patient NHS Numbers (a unique ten digit identifier). By linking the data between these two systems, iPlato will be able to identify individual-level screening episode data (return of test kit by the end of Week 7) from the Hub and individual’s mobile telephone number from the GP Clinical System. In order to have the best real life representation of overall screening uptake (intention-to-treat), all individuals with or without a mobile number are included in the study. iPlato will be using a secure NHS server (‘N3’) to bring the data together under one network. The iPlato system updates itself every 4 h, thereby removing in real time any individual from the trial who becomes removed from the GP system (e.g. due to reasons such as death or patient relocation).
3. Data Generation
  After 8 weeks of an individual’s screening episode, patients in the intervention group who have not returned their test kit and who have a mobile number stored on the GP Clinical System will be sent the text-message reminder. iPlato will generate several additional variables in response to this process, including: 1) whether a mobile record was available or not; 2) whether the individuals had already returned their test kit, and thereby whether a text-message was attempted; and 3) whether the text-message was successfully delivered or whether it failed (i.e. if the individual had an inactive mobile). Data Generation will be an on-going procedure each week until the sample size requirement is met.
4. Data Merge
  Once data collection is complete, iPlato will remove the mobile numbers and return the data to the Hub electronically through a secure N3 network connection. The Hub will add to the dataset whether the individual was adequately screened (yes/no) at the end of their individual episode (end of week 18), and then merge the dataset with any other variable that was not required by iPlato, but is useful for the purposes of the analysis (i.e. age, gender, episode number, social deprivation score, and adequately screened). The data merge will be a one-off procedure.
5. Anonymisation
  The researchers do not form part of the usual care team and as such will not have access to patient identifiable data at any point in the study. The Hub will remove all identifiable information from the dataset prior to analysis by the research team. Date of birth (DOB) will be converted into age at invite, gender encrypted using binary measures (i.e. 1 male, 2 female), and individual NHS numbers removed. Postcodes will be converted into scores based on census derived indicators of deprivation - i.e. the Index of Multiple Deprivation (IMD) [24]- and then removed from the dataset. As it is not required for analysis, the individuals GP code will be removed; however, the individuals CCG will be retained and will constitute one of the variables included in the multivariable analysis.
6. Extraction
  The anonymised dataset will be sent to the principal investigator.
7. Deletion
  Once the data have been extracted, anonymised, and transferred, the research team will confirm that the data collection process has been completed and that iPlato’s records of the data should be destroyed. The Hub will retain the anonymised data for three months and the researchers will store the anonymised data for 10 years.

### Sample size calculation

The study has been designed to detect a 5 percentage point increase in uptake between the intervention and control groups. The expected uptake of CRC screening in the control group (43.51 %) was based on the uptake in the selected CCGs in London between May and October 2014. The anticipated improvement in uptake in the intervention group (5 percentage points, to 48.51 %) was based on the results from similar studies evaluating the effectiveness of text-message reminders to facilitate uptake in other areas of healthcare [12]. To detect this increase in uptake with 80 % power and 5 % significance level, 1600 participants per trial arm are required, giving a total sample size of 3200 participants.

### Primary and secondary outcomes

The primary outcome is the return of an adequate test kit in all randomised participants (intention-to-treat analysis). Return of an adequate test kit will be defined as return of a gFOBt kit within 18 weeks of the invitation with a ‘definitive’ test result of either ‘normal’ (i.e. no further investigation required) or ‘abnormal’ (i.e. requiring referral for further testing, usually colonoscopy). Identification of individual-level mobile number registration on the GP Clinical System will constitute an important secondary outcome measure. Thereby, in order to test the efficacy of text-message reminders in CRC, we will limit the comparison of the effectiveness of a text reminder to those who have a registered mobile number on GPs Clinical System and have not returned their test kit within eight weeks (per-protocol analysis).

### Statistical analysis

The primary and secondary outcomes will be analysed using a univariable logistic regression model, and then adjusting for age, gender, IMD score, CCG and episode number (first episode vs incident episode) in a multivariable model. Sample characteristics will be reported using descriptive statistics. The comparison of overall uptake between trial arms will be presented using odds ratios (ORs) and 95 % confidence intervals (CIs).

### Ethical approval

This study has been reviewed by the East Midlands National Research Ethics Service, from which it received a favourable opinion (15/EM/0159).

### Informed consent

We will not seek consent from individuals because the intervention is embedded in a routine screening service and conducted through GPs with existing mobile health services. The study has been reviewed by the Confidentiality Advisory Group (CAG), and granted full approval (15/CAG/0156), permitting a Section 251 exemption for iPlato (the m-Health provider) to process patient identifiable information for the purposes of this study without prior consent from the individuals involved. However, we will recruit GPs that have been using m-health communications in order to ensure compliance with the NHS Information Governance Information Risk Management Guidance for Short Message Service (SMS) and Texting (2010) [25], The Good Practice Guidelines for GP electronic patient records v4 (2011) [26], and Data Protection Act 1998.

## Discussion

This study represents the first large-scale trial of a text-message reminder in the English BCSP. The key importance of this protocol is that it highlights a methodology for delivering text-message reminders in CRC screening through the involvement of primary care, which is both an opportunity and a challenge.

The opportunity of delivering a text-message reminder in this way relates to the value the public places on primary care involvement in organised screening [11]. A direct communication from primary care via a text reminder is therefore likely to benefit patients in their decision making by recommendation of bowel screening through a trusted source. In turn, if the text-message reminder is effective, the NHS (and Primary Care) would benefit by having to treat fewer late stage CRCs, due to an increase in uptake and, subsequently, screen-detected cancers [6].

The challenge arising from delivering text-message reminders in this way will depend upon the acceptance and willingness of healthcare professionals to adopt innovations in information technology [27, 28]. This will be evaluated after the recruitment process of the GPs, and the response rate will be useful in the future recruitment of GPs for research using m-health technologies.

Another important issue will be mobile phone coverage and more specifically the accuracy of the mobile phone records in primary care. Despite increased use of mobile phones in the general population, a recent study reported that only 39.8 % of the eligible population had a registered mobile on their GPs Clinical System [18]. This will also help inform the potential effectiveness & efficacy of a text-message reminder as a method of promoting CRC screening uptake in future studies.

If effective, this study provides a cost-effective means to promote uptake of CRC screening in an organised programme.
